# The association between metal element levels and thyroid nodules in oilfield workers: a cross-sectional study

**DOI:** 10.3389/fendo.2025.1590821

**Published:** 2025-05-20

**Authors:** Xinyue Wen, Feidan Deng, Lichun Qiao, Miaoqian Li, Xining Wang, Hongqiu Li, Huifang He, Yanjun Xie, Zhaoyang Li, Bowei Yang, Jing Han

**Affiliations:** ^1^ Ningxia Gem Flower Hospital, Health Management Center, Yinchuan, Ningxia, China; ^2^ Department of Occupational and Environmental Health, Key Laboratory of Environment and Genes Related to Diseases, School of Public Health, Health Science Center, Xi’an Jiaotong University, Xi’an, Shaanxi, China; ^3^ Key Laboratory for Disease Prevention and Control and Health Promotion of Shaanxi Province, Xi’an Jiaotong University, Xi’an, Shaanxi, China; ^4^ Global Health Institute, Health Science Center, Xi’an Jiaotong University, Xi’an, Shaanxi, China

**Keywords:** thyroid nodules, metal elements, iron, copper, oilfield worker

## Abstract

**Background:**

Metal elements affect the physiological processes of the thyroid gland and are associated with the formation of thyroid nodules (TNs). This study aimed to investigate the relationship between metal element levels and TNs in oilfield workers and to provide a preliminary scientific basis.

**Methods:**

The study used a cross-sectional study to collect relevant data in 2022. Spearman’s rank correlation was used to analyze the correlation between multiple metal elements. The Logistic regression model and Weighted Quantile Sum (WQS) regression model were used to analyze the association between metal elements and the prevalence of TNs.

**Results:**

A total of 517 oilfield workers were included in this study and the prevalence of TNs was 40.62%. Sex, age, and uric acid levels differed between the two groups (*P* < 0.05). The correlation analysis showed that most of the metals were correlated with each other to varying degrees. The WQS regression model showed that mixed exposure to seven metal elements was positively associated with the risk of developing TNs. In the total population and males, iron (Fe) and copper (Cu) levels were positively related to the risk of TNs prevalence (*P* < 0.05).

**Conclusions:**

TNs was found to be very prevalent among oilfield workers. Mixed exposure to metal elements may be associated with an elevated risk of TNs, with Fe and Cu emerging as potential contributors to this association.

## 1 Introduction

Thyroid nodules (TNs) are discrete lesions within the thyroid gland caused by abnormal, focal growth of thyroid cells. The imaging definition is a space-occupying lesion in the thyroid gland that can be detected by imaging and differentiated from the surrounding thyroid tissue (1). TNs may cause compressive symptoms (e.g., hoarseness, dyspnea, dysphagia) and disrupt thyroid hormone secretion, leading to hyperthyroidism or hypothyroidism, which significantly impair patients’ quality of life (2–5). Global epidemiological studies show that the prevalence of TNs is at a high level. A large Chinese study found that the overall prevalence of TNs was 36.9% (6). Studies conducted in Korea, Vietnam, the United States, Canada, South Africa, and Denmark showed that the prevalence of TNs was 34.2%, 48.8%, 30%, 30%, 79%, and 54%, respectively (7–9). Oilfield workers are at higher risk of TNs due to occupational factors such as night shift work (10) and occupational load (11). In China, the prevalence of TNs among oilfield workers in Jilin and petrochemical enterprise workers in Zhejiang reached 20.91% and 76.0%, respectively (12, 13).

Metal elements exert profound impacts on human health. Iron (Fe), a metal element essential for oxygen transport, is a critical component of hemoglobin and is indispensable for cellular energy metabolism and enzymatic reactions (14). Zinc (Zn) plays critical physiological roles, particularly in immune function and developmental processes (15). Copper (Cu) regulates diverse biological processes via redox activity and is implicated in the pathogenesis of multiple diseases (16). Calcium (Ca), the most copious mineral in the human body, fulfills structural and functional roles: approximately 99% of bodily calcium is stored in bones, while plasma Ca homeostasis regulates skeletal integrity, hormone secretion, neuronal signaling, and vascular function (17, 18). Magnesium (Mg) is indispensable for oxidative phosphorylation, glycolysis, and macromolecule synthesis (e.g., proteins, nucleic acids) (19). In contrast, heavy metals such as lead (Pb) and cadmium (Cd) exhibit toxicological effects, posing significant risks to human health (20).

Metal elements are essential for human life and critical to physiological processes, including the thyroid (21). In recent years, some scholars have studied the relationship between metal elements and TNs. A study from Guangdong, China, found that subjects with higher serum Zn, Mg, and Cu levels had a 1.23-fold, 1.04-fold, and 1.007-fold increased risk of thyroid nodule prevalence, respectively (*P* < 0.05) (22). Ma et al. identified Ca and Mg as significant TNs risk factors (23). Conversely, a Korean study reported a nonlinear dose-response relationship between Cu levels and TNs prevalence, with no associations observed for other metals (24). Kravchenko et al. demonstrated that reduced serum levels of Ca, Mg, Zn, Cu, and Pb correlated with increased nodular goiter risk (25).

Although studies have explored associations between metal elements and TNs, the evidence base remains inconclusive due to limited sample sizes and methodological heterogeneity. Notably, no prior research has systematically investigated this relationship in oilfield workers. Therefore, this study aims to investigate the relationship between metal elements and TNs in oilfield workers and to establish a foundational framework for exploring the prevention and treatment of TNs in occupational populations from the perspective of metal elements.

## 2 Materials and methods

### 2.1 Study subjects

**Figure 1:**
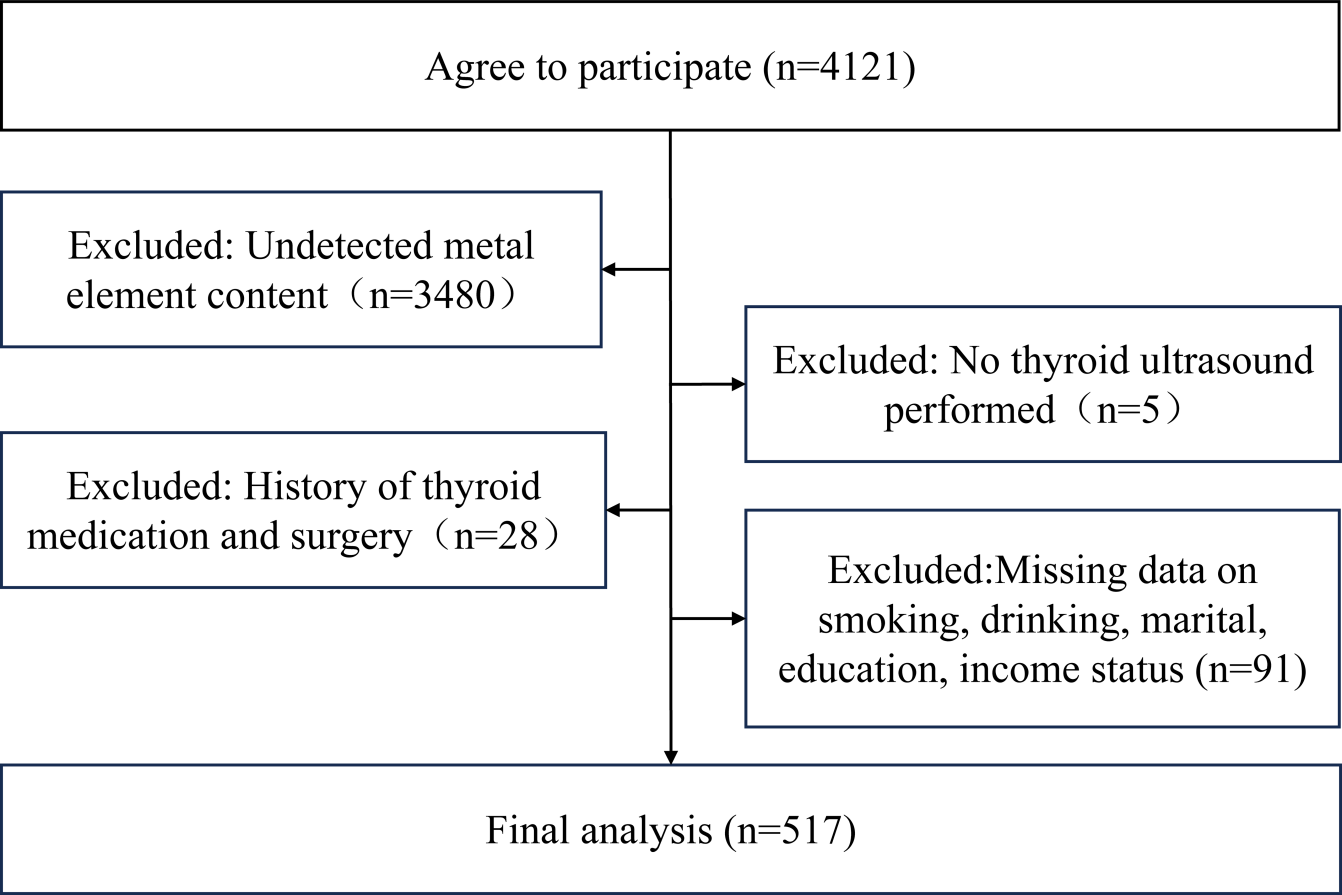
Flowchart of the inclusion and exclusion.

This study enrolled oilfield workers who underwent medical checkups between October and December 2022 at a designated hospital for oilfield workers in China. An on-site health questionnaire survey was administered to participants during their checkups, with data extracted from medical records. Informed consent was obtained from all participants prior to enrollment. The study protocol received ethical approval from the Biomedical Ethics Committee, Department of Medicine, Xi’an Jiaotong University (No. 2022-1539) and was conducted in accordance with institutional guidelines. The exclusion criteria implementation process was detailed in **Figure 1**. To ensure data integrity, we excluded study participants with missing basic information. Inclusion criteria: signed informed consent. Exclusion criteria: missing data, history of thyroid surgery and medication (26).

### 2.2 Questionnaire data collection

In this study, a structured questionnaire was used to conduct the field survey and all the researchers involved in the field work went through a systematic training. It mainly includes the following: basic information (name, sex, age, education, marital status, annual income status) (27, 28); occupational related factors (type of work, shift work, noise exposure, dust exposure); history of thyroid surgery; history of thyroid medication; smoking and drinking consumption status.

### 2.3 Anthropometric data collection

Anthropometric data such as height, weight, and body mass index (BMI) are measured by trained professionals using standardized measurement tools. The body mass index (BMI) = weight (kg)/height (m) squared (kg/m^2^) (29).

### 2.4 Laboratory analysis

All subjects fasted for more than 8 hours, and 5 ml of fasting venous blood was taken in the early morning for biochemical analysis. A biochemical automatic analyzer (AU-5800, Beckman Coulter, Brea, CA, USA) was used to measure total cholesterol (TC), triglycerides (TG), high-density lipoprotein (HDL), and low-density lipoprotein (LDL), fasting blood glucose (FBG), and uric acid (UA). Thyroid-stimulating hormone (TSH), free triiodothyronine (FT3), free thyroxine (FT4), total triiodothyronine (TT3), and total thyroxine (TT4) were measured by chemiluminescent immunoassay (Siemens, ADVIA Centaur XPT, Erlangen, Germany). Whole blood concentrations of selected metal elements Zn, Fe, Cu, Ca, Mg, Pb, and Cd were assessed by a trace element analyzer (Shanghai, China).

### 2.5 Color doppler ultrasound of the thyroid gland

Thyroid ultrasound was performed by an experienced sonographer using a high-frequency probe to observe the shape and size of the thyroid gland. If nodules were found, the number, size, morphology, and echogenicity of the nodules, borders, and the presence of calcifications were recorded.

### 2.6 Outcome definitions

The criteria for determining TNs refer to the Chinese Guidelines for the Diagnosis and Treatment of TNs and Differentiated Thyroid Cancer (Second Edition), and the imaging definition refers to the occupying lesions in the thyroid gland that can be detected by imaging and distinguished from the surrounding thyroid tissue. The presence of TNs is determined by ultrasound findings (1).

### 2.7 Statistical analysis

Continuous variables with normal distribution were expressed as mean ± standard deviation (SD) and compared using the independent samples t-test. Non-normally distributed variables were presented as median (interquartile range [IQR]) and analyzed with the Mann-Whitney U test. Categorical data were described as counts (n) with percentages (%), and group differences were assessed using Chi-square test.

Spearman’s rank correlation coefficient was used to analyze the correlation between seven metal elements. To account for magnitude differences, metal concentrations were naturally ln-transformed and standardized to z-scores (mean=0, SD=1) prior to analysis (30). The odds ratio (OR) thus represents the effect of a one-SD increase in metal exposure on TNs risk. Metals were analyzed as both continuous and categorical variables. Multivariable logistic regression models were fitted to evaluate single metal and multiple metal associations with TNs risk. The trend test was used to estimate the P-trend values for the tertiles of the seven metal elements by replacing the tertiles 1(T1), tertiles 2 (T2), and tertiles 3 (T3) exposure levels with the median levels of the metal elements in each group, which were treated as continuous variables in the model, to explore the trends in the prevalence of interstorey metal elements and TNs. Weighted Quantile Sum (WQS) regression modeling to analyze the effect of mixed exposure to metal elements on the risk of developing TNs. OR and 95% confidence intervals (CI) were calculated to assess TNs risk. All analyses were performed using SPSS 26.0 and R 4.3.3, with two-tailed statistical significance set at α=0.05.

## 3 Results

### 3.1 Baseline characteristics

A total of 517 oilfield workers were enrolled in this cross-sectional study, comprising 315 males (60.93%) and 202 females (39.07%) with a mean age of 49.12 ± 3.12 years. The prevalence of TNs was 40.62% (210/517). Males constituted a higher proportion of TNs cases (55.20%) compared to females (44.80%), with this sex difference reaching statistical significance (*P* < 0.05). Participants with TNs were significantly older than those non-TNs (*P* < 0.05), while serum UA levels were markedly lower in the TNs group (*P* < 0.05). No significant differences were observed between the groups regarding marital status, education level, annual income, smoking/drinking status, shift work, noise/dust exposure, BMI, TC, TG, HDL, LDL, FBG, or thyroid hormone levels (*P* > 0.05). Analysis of blood metals revealed significantly elevated Cu levels in the TNs group *(P* < 0.05), whereas Zn, Fe, Ca, Mg, Pb, and Cd levels showed no intergroup differences (*P* > 0.05) (**Supplementary Table S1**).

### 3.2 Correlation between the seven metal elements

**Figure 2:**
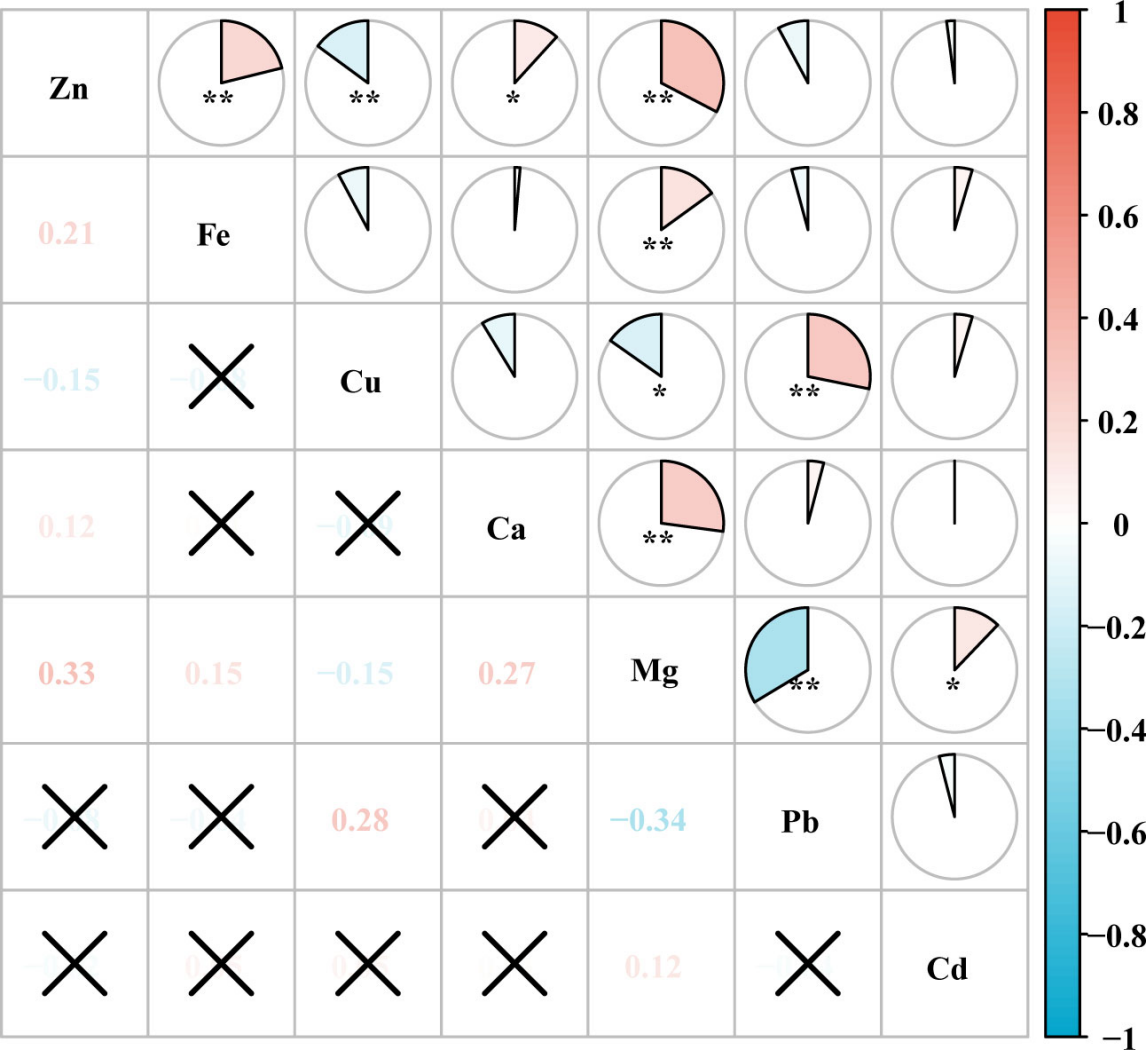
Heat map of correlation between metal elements. ^*^ *P* < 0.05, ^**^ *P* < 0.01. The numbers in the figure indicate the magnitude of the correlation coefficient. “✗” indicates that the correlation between the two metals is not significant.

The results of Spearman’s rank correlation analysis showed that after ln-transformed, most of the metals were correlated with each other to varying degrees. The results of correlation analysis showed significant correlations (*P* < 0.05) between Zn and Fe, Cu, Ca, Mg; Fe and Mg; Cu and Mg, Pb; Ca and Mg; Mg and Pb, Cd. The largest correlation coefficient was found between Mg and Pb, -0.34, followed by Mg and Zn with a correlation coefficient of 0.33 (**Figure 2**; **Supplementary Table S2**).

### 3.3 The association of single metal elements exposure with TNs

In Model 1 (unadjusted), Cu levels showed a statistically significant positive association with TNs risk (OR = 1.216, 95% CI: 1.016, 1.455; *P* < 0.05). No significant associations were observed for Zn, Fe, Ca, Mg, Pb, or Cd (*P* > 0.05). Model 2 adjusted for sex, age, BMI, income, education, smoking, and drinking status. Both Cu (OR = 1.206, 95% CI: 1.003, 1.450; *P* < 0.05) and Fe (OR = 1.223, 95% CI: 1.003, 1.491; *P* < 0.05) exhibited significant positive associations with TNs risk. Conversely, Zn, Ca, Mg, Pb, and Cd remained non-significant (*P* > 0.05) (**Table 1**).

**Table 1:** Effect of a single metal element on the risk of developing TNs.

Metal	Model 1		Model 2	
	OR (95%CI)	*P*	OR (95%CI)	*P*
Zn	1.011 (0.848, 1.205)	0.901	1.037 (0.865, 1.243)	0.694
Fe	1.068 (0.896, 1.274)	0.462	1.223 (1.003, 1.491)	0.046^*^
Cu	1.216 (1.016, 1.455)	0.033^*^	1.206 (1.003, 1.450)	0.046*
Ca	1.077 (0.904, 1.283)	0.409	1.052 (0.879, 1.259)	0.579
Mg	0.898 (0.754, 1.071)	0.233	0.882 (0.736, 1.058)	0.178
Pb	1.046 (0.876, 1.250)	0.618	1.090 (0.905, 1.313)	0.364
Cd	1.001 (0.840, 1.194)	0.988	1.029 (0.855, 1.238)	0.764

^*^ *P*<0.05; Model 1: not adjusted for any covariates; Model 2: adjusted for sex, age, BMI, income, education, smoking, and drinking status. Bolded values indicate statistical significance.

### 3.4 The association of multiple metal element exposure with TNs

#### 3.4.1 Logistic regression modeling to construct multiple metal models

In Model 1, Cu levels showed a positive association with TNs prevalence risk (OR = 1.233; 95% CI: 1.018, 1.492; *P* < 0.05), whereas Zn, Fe, Ca, Mg, Pb, and Cd exhibited no statistical significance (*P* > 0.05). In Model 2, Fe levels demonstrated a significant positive correlation with thyroid nodule development risk (OR = 1.253; 95% CI: 1.018, 1.543; *P* < 0.05), while Zn, Cu, Ca, Mg, Pb, and Cd showed no statistically significant associations (*P* > 0.05) (**Table 2**).

**Table 2:** Effect of multiple metal elements on the risk of developing TNs.

Metal	Model 1		Model 2	
	OR (95%CI)	*P*	OR (95%CI)	*P*
Zn	1.056 (0.872, 1.278)	0.579	1.068 (0.876, 1.301)	0.517
Fe	1.090 (0.908, 1.310)	0.355	1.253 (1.018, 1.543)	0.033^*^
Cu	1.233 (1.018, 1.492)	0.032^*^	1.205 (0.989, 1.466)	0.064
Ca	1.134 (0.942, 1.365)	0, 184	1.116 (0.922, 1.351)	0.261
Mg	0.857 (0.698, 1.051)	0.139	0.828 (0.669, 1.024)	0.082
Pb	0.942 (0.776, 1.145)	0.549	0.985 (0.804, 1.206)	0.883
Cd	1.004 (0.839, 1.202)	0.964	1.032 (0.854, 1.248)	0.743

^*^ *P*<0.05; Model 1: not adjusted for any covariates; Model 2: adjusted for sex, age, BMI, income, education, smoking, and drinking status. Bolded values indicate statistical significance.

#### 3.4.2 WQS regression modeling for modeling mixed multiple metal exposures

**Figure 3:**
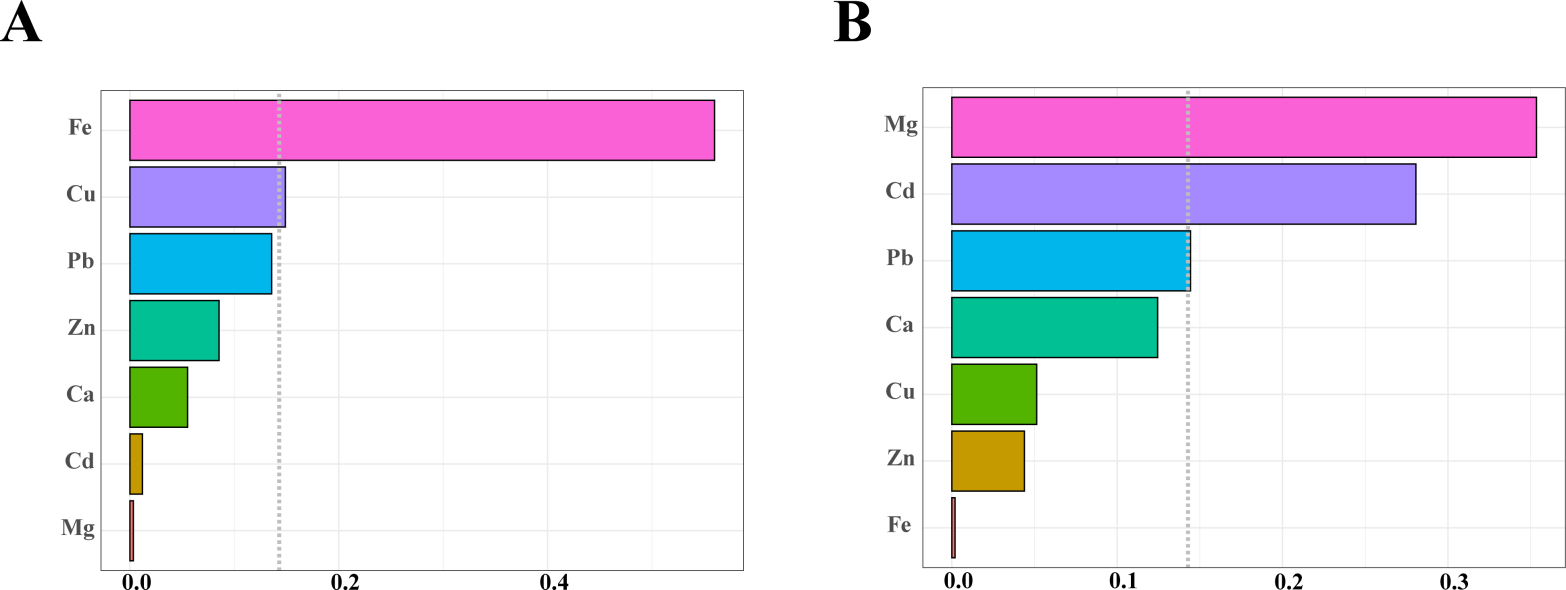
Estimated weights of each metal elements in the WQS regression model. **(A)** Positive weight distribution graph. **(B)** Negative weight distribution graph. The dotted line indicated the position of the reference value, and metal elements with estimated weights exceeding the reference value were considered to have a significant effect on the WQS index.

The WQS regression analysis demonstrated that under positive-direction constraints, the seven metal elements were significantly associated with an elevated risk of TNs (OR = 1.870, 95% CI: 1.111, 3.144, *P* < 0.05). When restricted to negative directionality, the outcome showed no statistical significance (OR = 1.420, 95% CI: 0.783, 2.577, *P* > 0.05) (**Table 3**). Weight distributions of seven metals derived from 10,000 bootstrap iterations (**Figure 3**). Metal elements with estimated weights greater than 14.3% (1/7) were considered to have a significant effect on the WQS index. Under positive-direction constraints, Fe (56.0%) and Cu (14.9%) were weighted more than 14.3% (**Figure 3A**, **Table 4**). Under negative-direction constraints, Mg (35.4%), Cd (28.0%), and Pb (14.4%) were weighted more than 14.3% (**Figure 3B**, **Table 4**). Since the WQS results only found a significant positive association between mixed metals and the risk of developing TNs, Fe and Cu were the two important metal elements that influence the risk of developing TNs.

**Table 3:** Mixed exposure effects of seven metal elements.

WQS	OR (95%CI)	*P*
Positive	1.870 (1.111, 3.144)	0.017^*^
Negative	1.420 (0.783, 2.577)	0.247

^*^ *P*<0.05; Model adjusted for sex, age, BMI, income, education, smoking, and drinking status. Bolded values indicate statistical significance.

**Table 4:** Weights of each metal element in the WQS regression model.

Metal	Positive weights	Negative weights
Zn	0.085	0.044
Fe	0.560	0.002
Cu	0.149	0.051
Ca	0.055	0.124
Mg	0.003	0.354
Pb	0.136	0.144
Cd	0.012	0.280

### 3.5 The association of Fe and Cu with TNs

When Fe and Cu were analyzed as continuous variables, Model 1 demonstrated a significant positive association between Cu levels and TNs prevalence (OR = 1.221; 95% CI: 1.020, 1.462; *P* < 0.05), whereas no such association was detected for Fe (*P* > 0.05). This association pattern persisted in Model 2, with Cu showing a comparable effect size (OR = 1.213; 95% CI: 1.008, 1.460; *P* < 0.05). Notably, elevated Fe levels exhibited a significant association with TNs (OR = 1.231; 95% CI: 1.008, 1.504; *P* < 0.05) (**Table 5**).

**Table 5:** The association of Fe and Cu with TNs.

Metal	Model 1		Model 2	
	OR (95%CI)	*P*	OR (95%CI)	*P*
Fe	1.079 (0.904, 1.288)	0.400	1.231 (1.008, 1.504)	0.041^*^
T1	Ref.		Ref.	
T2	1.051 (0.680, 1.623)	0.824	1.165 (0.736, 1.844)	0.514
T3	1.296 (0.842, 1.994)	0.239	1.771 (1.089, 2.879)	0.021^*^
*P-trend*	0.242		0.021^*^	
Cu	1.221 (1.020, 1.462)	0.030^*^	1.213 (1.008, 1.460)	0.041^*^
T1	Ref.		Ref.	
T2	1.053 (0.681, 1.630)	0.816	1.031 (0.658, 1.617)	0.893
T3	1.438 (0.934, 2.215)	0.099	1.355 (0.866, 2.119)	0.184
*P-trend*	0.102		0.215	

^*^ *P*<0.05; Model 1: not adjusted for any covariates; Model 2: adjusted for sex, age, BMI, income, education, smoking, and drinking status. Bolded values indicate statistical significance.

When analyzed as categorical variables, neither metal was significantly associated with TNs in Model 1 (*P* > 0.05). However, Model 2 revealed a 77.1% increased TNs risk for participants in the highest Fe T3 compared to the lowest T1 (OR = 1.771; 95% CI: 1.089, 2.879; *P* < 0.05). No significant association emerged for Cu in this model. The trend test showed that the risk of prevalence of TNs increased with increasing Fe levels (*P-trend*=0.021) (**Table 5**).

### 3.6 Sex-stratified analysis of the relationship between Fe and Cu and TNs

In males, when Fe and Cu were analyzed as continuous variables, both elements showed significant positive associations with TNs risk in Model 1 (Fe: OR = 1.286, 95% CI: 1.007, 1.643, *P* < 0.05; Cu: OR = 1.289, 95% CI: 1.002, 1.657, *P* < 0.05) and Model 2 (Fe: OR = 1.349, 95% CI: 1.043, 1.746, *P* < 0.05; Cu: OR = 1.328, 95% CI: 1.023, 1.724, *P* < 0.05). When analyzed as categorical variables, significant associations were observed in Model 1 for Cu (T3 vs T1: OR = 2.252, 95% CI: 1.249, 4.063, *P* < 0.05), though Fe showed no association in Model 1 (*P* > 0.05). In Model 2, both Fe (T3 vs T1: OR = 1.980, 95% CI: 1.022, 3.836, *P* < 0.05) and Cu (T3 vs T1: OR = 2.165, 95% CI: 1.172, 3.999, *P* < 0.05) showed significant positive associations with TNs. The trend test showed that the risk of prevalence of TNs increased with increasing Fe (*P-trend*= 0.040 in Model 1; 0.025 in Model 2) and Cu (*P-trend* = 0.006 in Model 1; 0.013 in Model 2) (**Table 6**).

**Table 6:** Sex-stratified analysis of the association of Fe and Cu with TNs.

Metal	Model 1		Model 2	
	OR (95%CI)	*P*	OR (95%CI)	*P*
Males				
Fe	1.286 (1.007, 1.643)	0.044^*^	1.349 (1.043, 1.746)	0.023^*^
T1	Ref.		Ref.	
T2	1.179 (0.603, 2.304)	0.631	1.158 (0.577, 2.322)	0.680
T3	1.828 (0.975, 3.426)	0.060	1.980 (1.022, 3.836)	0.043^*^
*P-trend*	0.040^*^		0.025^*^	
Cu	1.289 (1.002, 1.657)	0.048^*^	1.328 (1.023, 1.724)	0.033^*^
T1	Ref.		Ref.	
T2	1.646 (0.933, 2.906)	0.086	1.584 (0.883, 2.841)	0.123
T3	2.252 (1.249, 4.063)	0.007^**^	2.165 (1.172, 3.999)	0.014^*^
*P-trend*	0.006^**^		0.013^*^	
Females				
Fe	1.022 (0.740, 1.411)	0.897	0.966 (0.687, 1.357)	0.841
T1	Ref.		Ref.	
T2	1.236 (0.663, 2.305)	0.505	1.162 (0.599, 2.256)	0.657
T3	1.177 (0.517, 2.678)	0.698	0.905 (0.380, 2.153)	0.821
*P-trend*	0.508		0.876	
Cu	1.107 (0.851, 1.440)	0.449	1.102 (0.838, 1.450)	0.486
T1	Ref.		Ref.	
T2	0.531 (0.256, 1.101)	0.089	0.419 (0.191, 0.918)	0.030^*^
T3	0.698 (0.358, 1.361)	0.291	0.672 (0.332, 1.359)	0.269
*P-trend*	0.274		0.234	

^*^ *P*<0.05, ^**^ *P*<0.01; Model 1: not adjusted for any covariates; Model 2: adjusted for gender, age, BMI, income, education, smoking, and alcohol status. Bolded values indicate statistical significance.

In females, neither continuous nor categorical analyses revealed significant associations for Fe (*P* > 0.05). Notably, Cu in T2 showed protective effects against TNs compared to T1 in Model 2 (OR = 0.419, 95% CI: 0.191, 0.918, *P* < 0.05), though no significant trends were detected (*P-trend* > 0.05) (**Table 6**).

## 4 Discussion

This cross-sectional study investigated associations between seven metal elements (Zn, Fe, Cu, Ca, Mg, Pb, Cd) and TNs risk among 517 oilfield workers. Comprehensive data including demographic characteristics, physical/laboratory examinations, and thyroid ultrasound findings were collected. The overall TNs prevalence was 40.62%, with significant differences observed between TNs and non-TNs groups in sex, age, and UA levels. Mixed exposure to the seven metals demonstrated a positive association with TNs risk. Specifically, both Fe and Cu levels showed significant positive associations in the total population, with consistent patterns observed in male subgroups.

The results of this study showed that the prevalence of TNs among these oilfield workers was 40.62%, which was higher than the global prevalence of TNs in the general population (24.83%) and higher than the prevalence of TNs in the most recent report of a large health screening cohort in China (36.9%) (8, 31). In addition, the prevalence of TNs in this population was higher than the local level when compared to the prevalence of TNs in the general population where the samples were collected (26.4%) (6). This difference may be due to the specificity of the occupation, where oilfield workers may be exposed to more factors that contribute to the development of TNs. Compared with the previously reported prevalence of TNs in the oilfield occupational population, it was higher than that of Jilin oilfield workers (20.91%) and lower than that of Zhejiang petrochemical enterprise workers (76.0%) (12, 13). This difference may stem from the difference in ultrasound methods; both this study and the Zhejiang study used color Doppler ultrasound for examination, whereas the Jilin oilfield used Gray-scale ultrasound, and color Doppler ultrasound had a higher sensitivity than Gray-scale ultrasound (32, 33). Thus, the prevalence of TNs reported in the Jilin oilfields was relatively low.

Sex is an important factor influencing the occurrence of TNs, and in the present study, it was found that females accounted for 44.80% of patients with TNs, which was higher than 35.18% of non-TNs patients. Females are affected by sex-related hormones such as physiology, pregnancy, estrogen, and progesterone (34). Estrogen promotes the proliferation of thyroid stem cells through classical genomic and non-genomic pathways and leads to the development of TNs (35, 36). In addition, age is an important factor influencing TNs, and previous studies have shown that the prevalence of TNs increases with age (37). Our findings showed that the mean age in TNs group (49.45 ± 3.67) was higher than the mean age in non-TNs (48.90 ± 3.79), which is consistent with the results of previous studies. Our analysis showed that UA levels were statistically different between the two groups, with the TNs group having lower UA levels than non-TNs. Previous findings have been inconsistent, with some studies finding UA to be an independent risk factor for the formation of TNs, while others have found UA to be a protective factor for TNs in males over 30 years of age, which still needs to be further explored (38, 39).

The results of this study showed that Fe levels were positively correlated with the risk of developing TNs in the total population and males, and the risk of developing TNs increased with increasing Fe levels. This is inconsistent with the findings of Ma et al, in whose study no association between Fe and TNs was observed (23). There are fewer relevant studies and limited evidence. Fe influences the risk of developing TNs may be related to thyroid hormones. Excess Fe can generate large amounts of reactive oxygen species (ROS) by participating in electron transfer in the oxidative respiratory chain in the mitochondria, and excess ROS subsequently leads to mitochondrial dysfunction, oxidative stress, lipid peroxidation, and DNA damage, which ultimately leads to Fe-dependent cell death, which in turn affects the normal functioning of the thyroid gland (40). Fe is also involved in the regulation of the immune system. Chronic inflammation is one of the important pathogenetic mechanisms of TNs, and Fe overload may promote the formation of TNs by inducing chronic inflammation (41).

The results of this study showed that Cu levels were positively associated with the risk of TNs in the total population and males and that the risk of TNs increased with increasing Cu levels. Zeng et al. showed that subjects with higher Cu levels had an increased risk of thyroid nodule prevalence (*P* < 0.001), and participants in the fourth quartile had the highest prevalence of TNs among all participants compared to the first quartile of Cu levels in serum, which is in agreement with our study results (22). However, in another study, Cu levels were found to be unrelated to the development of nodular goiter (42). It has been proposed that the MAPK signaling pathway involved in cell proliferation is stimulated by Cu and that the cellular influx of Cu also enhances the phosphorylation of ERK1/2 through the interaction of Cu with MEK1, and thus higher levels of Cu may be involved in the pathogenesis of TNs through Cu-MEK1 interaction (22). Despite these insights, the precise molecular mechanisms linking Cu homeostasis to TNs pathogenesis remain poorly characterized, warranting further investigation.

However, this study also has some limitations. First, this study is a cross-sectional survey, and the results of the study can only be used to make a preliminary judgment of whether they are related or not, and cannot be used for causal argumentation; therefore, more longitudinal data need to be collected and prospective cohort studies need to be carried out to understand the causal relationship. Second, the restricted sample size from a specialized occupational cohort (oilfield workers) limits statistical power and generalizability. Extrapolation to general populations requires confirmation in multi-center studies encompassing diverse occupational exposures. Third, our study did not consider the effects of dietary structure, iodine status, genetics and other confounding factors on TNs and Metal elements. Future investigations should incorporate these covariates using standardized nutritional assessments and genome-wide association approaches.

## 5 Conclusions

Our study found that TNs were very prevalent among oilfield workers. Mixed exposure to metal elements may be associated with an elevated risk of TNs, with Fe and Cu emerging as potential contributors to this association. However, given the inherent limitations of cross-sectional designs in establishing causality, these results should be interpreted as exploratory evidence highlighting prioritized metals for further investigation. Future prospective studies are needed to verify causality, which will help develop targeted prevention strategies for this occupational group.

## Data Availability

The raw data supporting the conclusions of this article will be made available by the authors, without undue reservation.
