# Supplementary material for: The association between metal element levels and thyroid nodules in oilfield workers: a cross-sectional study
Source: Front Endocrinol (Lausanne). 2025 May 20;16:1590821. doi: 10.3389/fendo.2025.1590821 (PMC12129806; doi:10.3389/fendo.2025.1590821)
Supplement: Supplementary file 1 [file Table1.docx]

Supplementary Material

# Supplementary Data

**Table S1.** Comparison of demographic characteristics of oilfield workers grouped according to their prevalence of TNs

| **Characteristic** | **Overall** | **TNs** | | ***P*** |
| --- | --- | --- | --- | --- |
|  |  | **Yes** | **No** |  |
| Number | 517 | 210(40.62) | 307(59.38) |  |
| Sex, n(%) |  |  |  | **0.028^*^** |
| Male | 315(60.93) | 116(55.20) | 199(64.82) |  |
| Female | 202(39.07) | 94(44.80) | 108(35.18) |  |
| Age (years) | 49.12 ± 3.12 | 49.45 ± 3.67 | 48.90 ± 3.79 | **0.032^*^** |
| Marital status, n(%) |  |  |  | 0.469 |
| Married | 472(91.30) | 194(92.38) | 278(90.55) |  |
| Divorced | 45(8.70) | 16(7.62) | 29(9.45) |  |
| Education, n(%) |  |  |  | 0.705 |
| High school below | 112(21.66) | 43(20.48) | 69(22.48) |  |
| High school | 184(35.59) | 79(37.62) | 105(34.20) |  |
| High school up | 221(42.75) | 88(41.90) | 133(43.32) |  |
| Annual income, 10,000, n(%) |  |  |  | 0.160 |
| ≤10 | 124(24.98) | 55(26.19) | 69(22.48) |  |
| 11-15 | 316(61.12) | 131(62.38) | 185(60.26) |  |
| ≥16 | 77(13.99) | 24(11.43) | 53(17.26) |  |
| Smoking status, n(%) |  |  |  | 0.628 |
| Non-smoker | 274(53.00) | 114(54.29) | 160(52.12) |  |
| smoker | 243(47.00) | 96(45.71) | 147(47.88) |  |
| Drinking status, n(%) |  |  |  | 0.580 |

**Table S1.** Continued

| **Characteristic** | **Overall** | **TNs** | | ***P*** |
| --- | --- | --- | --- | --- |
|  |  | **Yes** | **No** |  |
| Non-drinker | 325(62.86) | 135(64.29) | 190(61.89) |  |
| Current drinker | 192(37.14) | 75(35.71) | 117(38.11) |  |
| Type of work, n(%) |  |  |  | 0.886 |
| oil recovery post | 290(56.09) | 117(55.71) | 173(56.35) |  |
| Non-oil recovery post | 227(43.91) | 93(44.29) | 134(43.65) |  |
| Shift work, n(%) |  |  |  | 0.479 |
| Yes | 313(60.54) | 131(62.38) | 182(59.28) |  |
| No | 204(39.46) | 79(37.62) | 125(40.72) |  |
| Noise, n(%) |  |  |  | 0.394 |
| Yes | 316(61.12) | 133(63.33) | 183(59.61) |  |
| No | 201(38.88) | 77(36.67) | 124(40.39) |  |
| Dust, n(%) |  |  |  | 0.365 |
| Yes | 134(25.92) | 50(23.81) | 84(27.36) |  |
| No | 383(74.08) | 160(76.19) | 223(72.64) |  |
| BMI (kg/m²) | 24.10(22.40, 26.30) | 24.25(22.60, 26.40) | 24.07(22.20, 26.10) | 0.247 |
| TC (mmol/L) | 4.47 ± 0.88 | 4.50 ± 0.95 | 4.46 ± 0.83 | 0.589 |
| TG (mmol/L) | 1.54(1.17, 2.25) | 1.50(1.15, 2.19) | 1.61(1.17, 2.30) | 0.387 |
| HDL (mmol/L) | 1.20(1.02, 1.36) | 1.19(1.03, 1.37) | 1.20(1.02, 1.36) | 0.789 |
| LDL (mmol/L) | 2.67 ± 0.70 | 2.70 ± 0.75 | 2.65 ± 0.66 | 0.378 |
| FBG (mmol/L) | 4.99(4.67, 5.38) | 4.99(4.66, 5.44) | 5.00(4.70,5.37) | 0.633 |
| UA (umol/L) | 333.20(267.90,385.25) | 323.85(257.00, 371.45) | 339.80(274.10, 394.70) | **0.006^*^** |
| Zn (mg/L) | 7.94(7.04,8.83) | 7.90(7.00,8.81) | 7.94(7.08,8.84) | 0.751 |
| Fe (mg/L) | 484.18(445.08, 523.55) | 489.48(443.55, 529.41) | 483.06(445.64, 521.59) | 0.397 |
| Cu (mg/L) | 0.96(0.74, 1.19) | 1.01(0.77, 1.22) | 0.93(0.71, 1.15) | **0.023^*^** |
| Ca (mg/L) | 76.95(69.74, 85.17) | 77.75(69.74, 86.57) | 76.15(69.74, 83.76) | 0.441 |
| Mg (mg/L) | 24.55(20.66, 28.44) | 24.55(20.17, 28.44) | 24.79(21.15, 28.92) | 0.255 |
| Pb (µg/ L) | 37.30(22.12, 65.34) | 38.34(23.83, 65.34) | 36.83(20.76, 65.46) | 0.583 |
| Cd (µg/L) | 0.03(0.02, 0.04) | 0.03(0.02, 0.04) | 0.03(0.02, 0.04) | 0.767 |

**Table S1.** Continued

| **Characteristic** | **Overall** | **TNs** | | ***P*** |
| --- | --- | --- | --- | --- |
|  |  | **Yes** | **No** |  |
| TT4 (nmol/L) | 121.72(107.37, 134.64) | 120.95(107.32, 133.53) | 122.11(107.34, 134.79) | 0.924 |
| TT3(nmol/L) | 1.61(1.44, 1.78) | 1.60(1.45, 1.79) | 1.61(1.43, 1.78) | 0.430 |
| FT3(pmol/L) | 5.36(5.05, 5.78) | 5.35(4.98, 5.76) | 5.41(5.08, 5.83) | 0.115 |
| FT4 (pmol/L) | 11.31(10.40, 12.41) | 11.23(10.47, 12.19) | 11.40(10.38, 12.61) | 0.385 |
| TSH (ulU/mL) | 2.13(1.55, 3.12) | 2.20(1.57, 3.23) | 2.09(1.54, 3.12) | 0.604 |

Note: ^*^*P*<0.05; Mean ± standard deviation indicates normally distributed continuous variables; median and interquartile spacing indicate interquartile range [IQR] continuous variables that do not fit the normal distribution; frequency (composition ratio) indicates categorical variables.

**Table S2** Correlation between metal elements

| **Metal** | **Zn** | **Fe** | **Cu** | **Ca** | **Mg** | **Pb** | **Cd** |
| --- | --- | --- | --- | --- | --- | --- | --- |
| Zn | 1.00 |  |  |  |  |  |  |
| Fe | 0.21^**^ | 1.00 |  |  |  |  |  |
| Cu | -0.15^**^ | -0.08 | 1.00 |  |  |  |  |
| Ca | 0.12^*^ | 0.01 | -0.09 | 1.00 |  |  |  |
| Mg | 0.33^**^ | 0.15^**^ | -0.15^*^ | 0.27^**^ | 1.00 |  |  |
| Pb | -0.08 | -0.04 | 0.28^**^ | 0.04 | -0.34^**^ | 1.00 |  |
| Cd | -0.02 | 0.05 | 0.05 | 0.01 | 0.12^*^ | -0.04 | 1.00 |

Note：^*^*P*<0.05，^**^*P*<0.01. The numbers indicate the values of the correlation coefficients.
